# Synthetic lethality of a cell-penetrating anti-RAD51 antibody in PTEN-deficient melanoma and glioma cells

**DOI:** 10.18632/oncotarget.26654

**Published:** 2019-02-12

**Authors:** Audrey Turchick, Yanfeng Liu, Weixi Zhao, Inessa Cohen, Peter M. Glazer

**Affiliations:** ^1^ Department of Genetics, Yale University School of Medicine, New Haven, CT, USA; ^2^ Department of Therapeutic Radiology, Yale University School of Medicine, New Haven, CT, USA

**Keywords:** DNA repair, PTEN, RAD51, ATR, 3E10

## Abstract

PTEN is a tumor suppressor that is highly mutated in a variety of human cancers. Recent studies have suggested a link between PTEN loss and deficiency in the non-homologous end-joining (NHEJ) pathway of DNA double strand break (DSB) repair. As a means to achieve synthetic lethality in this context, we tested the effect of 3E10, a cell-penetrating autoantibody that inhibits RAD51, a key factor in the alternative pathway of DSB repair, homology dependent repair (HDR). We report here that treatment of PTEN-deficient glioma cells with 3E10 leads to an accumulation of DNA damage causing decreased proliferation and increased cell death compared to isogenic PTEN proficient controls. Similarly, 3E10 was synthetically lethal to a series of PTEN-deficient, patient-derived primary melanoma cell populations. Further, 3E10 was found to synergize with a small molecule inhibitor of the ataxia telangiectasia and Rad3-related (ATR) protein, a DNA damage checkpoint kinase, in both PTEN-deficient glioma cells and primary melanoma cells. These results point to a targeted synthetic lethal strategy to treat PTEN-deficient cancers through a combination designed to disrupt both DNA repair and DNA damage checkpoint signaling.

## INTRODUCTION

Phosphatase and tensin homolog (PTEN) is a tumor suppressor known to negatively regulate the phosphoinositide 3-kinase (PI3K)/AKT signaling axis in order to control cell cycle progression, growth, and survival [1]. PTEN is frequently mutated or lost through chromosomal deletion in various human cancers, notably glioblastomas, melanomas and prostate cancers [2-8]. Further, loss of PTEN is often associated with higher grade tumors and shorter progression free and overall survival [9, 10]

PTEN loss has also been associated with chromosomal instability, sensitivity to DNA damaging agents, and compromised genomic integrity [11-15]. Many publications have linked PTEN to DNA double strand break (DSB) repair [13, 16-18]. DNA double-strand breaks (DSBs) are the most deleterious form of DNA damage, but DSBs are repaired by two main pathways: non-homologous end-joining (NHEJ) and homology-directed repair (HDR). PTEN has been implicated to play a role in both HDR and NHEJ, but recently, our group has reported that PTEN promotes NHEJ by epigenetically inducing *XLF* gene expression [18], such that PTEN null cells show reduced XLF expression and consequently diminished NHEJ efficiency.

Recently, there has been an increasing focus on the therapeutic exploitation of DNA repair pathways for cancer therapy [19-21]. One example of this is the application of poly(ADP) ribose polymerase (PARP) inhibitors to selectively kill cancer cells with HDR deficiency. Patients with mutations in BRCA1 and BRCA2 have been successfully treated in clinical trials with PARP inhibitors, leading to recent regulatory approvals. Recently, investigators have expanded clinical trials of PARP inhibitors to include malignancies with mutations in or deficiency of PTEN [22] (https://clinicaltrials.gov/ ID NCT02286687).

Numerous other pharmacological strategies are being advanced to inhibit DNA repair, and most utilize small molecules. As an alternative, our group has recently discovered that treatment of human cells with the cell-penetrating autoantibody, 3E10, inhibits DNA DSB repair by HDR through a physical interaction between 3E10 and RAD51 [23]. We demonstrated that 3E10 inhibits RAD51 accumulation on ssDNA and RAD51-dependent DNA strand exchange. Further, 3E10 inhibits RAD51 foci formation in response to ionizing radiation or etoposide.

Loss of PTEN also leads to replication stress, and He and colleagues suggest that the PTEN-RAD51 signaling axis acts in response to replication stress to ensure successful DNA replication [24]. RAD51 is known to be a key player at stalled replication forks and for repair of DNA breaks at collapsed forks. If stalled replication forks are intact, XRCC3 and RAD51-mediated strand invasion have been shown to support fork restart [25]. However, in the case of collapsed replication forks, new origin firing is required to rescue replication, and repair of the collapsed forks is dependent on classical RAD51-mediated HDR [25]. Because RAD51 is critical for successful replication in PTEN deficient cells, and since 3E10 inhibits HDR through an interaction with RAD51, we hypothesized that cells deficient in PTEN would not only have reduced DNA DSB repair *via* NHEJ, but would also have excessive replication stress, and thus increased sensitivity to RAD51 inhibition by 3E10.

Further, the ataxia telangiectasia-mutated- and Rad3-related (ATR) kinase is recruited to replication protein A (RPA) coated single-stranded DNA at stalled replication forks and sites of DNA damage [26]. ATR mediated activation of the CHK1 protein leads to a signaling cascade and checkpoint response that protects cells from replication stress and ensures genomic integrity is maintained through proper replication fork progression [26, 27]. Thus, ATR is a critical component of replicating cells and has proven to be an attractive target for small molecule inhibition. Additionally, a recent study demonstrated the potential therapeutic benefit of an ATR inhibitor (VE-821) in PTEN-deficient breast cancers [28]. Because of this, we hypothesized that cells deficient in PTEN would also be sensitive to the combination of 3E10 and an ATR inhibitor (VE-822).

Here we report that 3E10 affects cellular viability of PTEN deficient cells in both glioma cell lines and in patient-derived primary melanoma cultures, indicating that inhibiting HDR with 3E10 leads to cytotoxicity in PTEN deficient cells. PTEN deficient cells treated with 3E10 have an increased burden of DNA damage, shown by an accumulation of DNA repair foci and micronuclei. This increased DNA damage confers synergism with an ATR inhibitor in both glioma and melanoma cells. Together this provides evidence to develop targeted synthetic lethal approaches in PTEN-deficient cancers through combination therapies that will further aid in the development personalized treatment strategies.

## RESULTS

### 3E10 scFv confers synthetic lethality with PTEN deficiency in a glioma cell line model system

**Figure 1:**
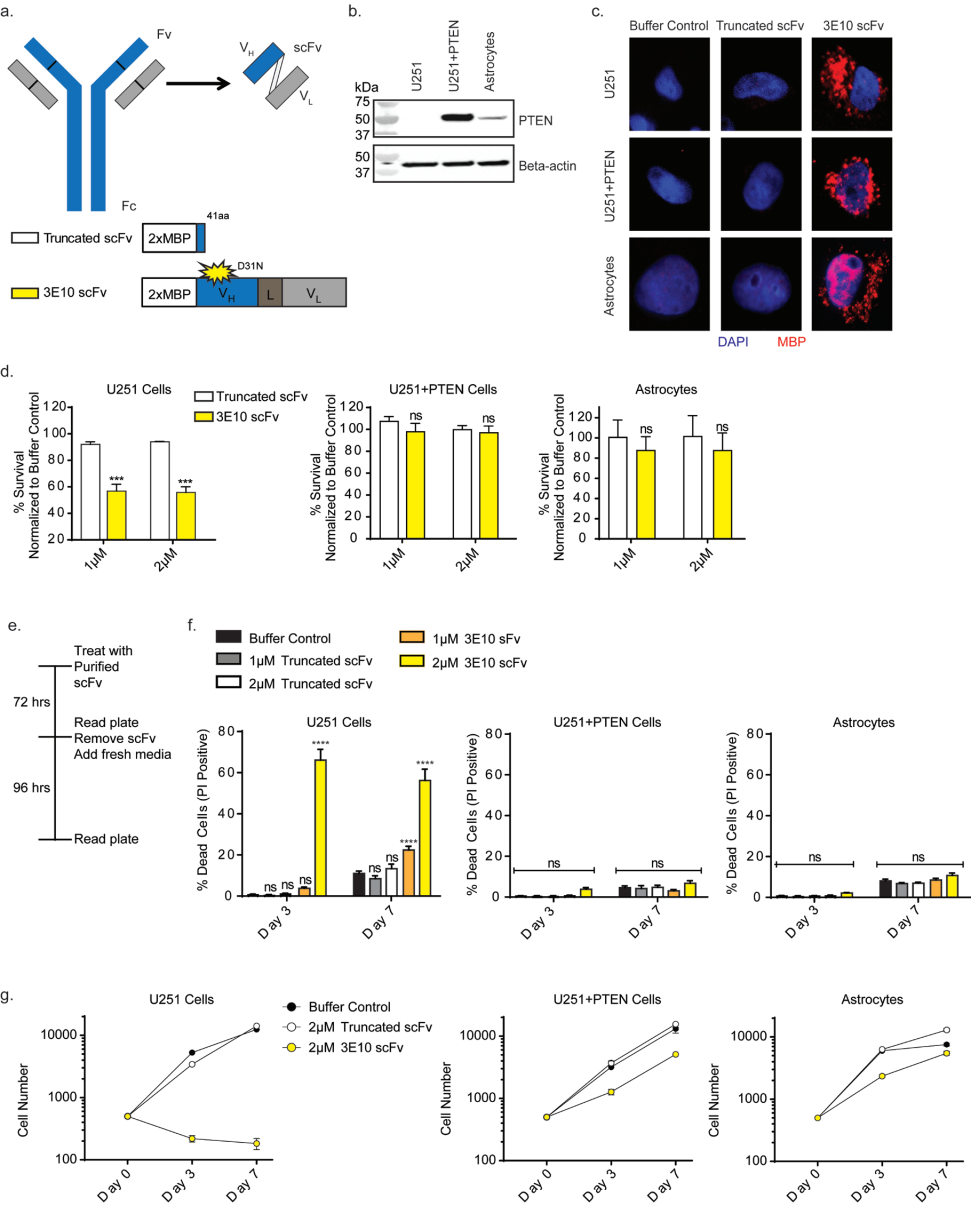
3E10 scFv confers synthetic lethality with PTEN deficiency in a glioma cell line **a.** Schematic of the 3E10 full length antibody as well as the derived scFv. The scFv consists of the heavy chain (V_H_) and light chain (V_L_) connected by a flexible linker (L). The scFv was cloned into a 2XMBP_phCMV1 expression vector. Expression constructs for each scFv are schematized. **b.** Western blot analysis of PTEN expression in U251 glioma cells, the PTEN complemented U251+PTEN cells and human astrocyte cells. **c.** Immunofluorescence analysis of cellular uptake of the purified scFv proteins in the U251, U251+PTEN and human astrocyte cells. **d.** Clonogenic survival results of U251, U251+PTEN or astrocyte cell lines treated with purified 3E10-scFv proteins. Error bars represent the SEM; ***P < 0.001 by unpaired *t*-test. **e.** U251, U251+PTEN or astrocyte cell lines were treated with either truncated scFv or 3E10 scFv at 1 μM or 2 μM for three days and then grown in fresh media for an additional four days. On day 3 and day 7, cells were stained with Hoescht dye and propidium iodide in order to quantify cell death over time. **f.** Cell death for each cell line under each treatment condition was plotted for each time point. Error bars represent the SEM; ****P < 0.0001 by unpaired *t*-test. **g.** Representative cell number for each cell line under each treatment condition was plotted for each time point.

We recently reported that 3E10 inhibits HDR and does so through a physical interaction with RAD51, resulting in a functional RAD51 inhibition [23]. Based on prior work suggesting that PTEN loss causes a reduction in NHEJ, the other major cellular pathway of DNA DSB repair [18], we sought to test the effect of the 3E10 on PTEN deficient cells. To minimize the risk of non-specific antibody-dependent cell-mediated cytotoxicity (ADCC) *via* the Fc region of 3E10, we focused on the single-chain variable fragment (scFv) rather than the full length antibody. Both the full-length 3E10 antibody and a scFv penetrate cells and show anti-RAD51 activity. The scFv construct we used contains the variable region of the heavy chain connected to the variable region of the light chain by a flexible linker, as schematized in Figure 1a. along with two tandem repeats of the maltose binding protein tag (2XMBP) at the N-terminus [23]. The MBP tags serve to facilitate affinity purification as well as to enhance solubility and stability of the recombinant antibodies [29]. We tested two versions of the 3E10 scFv. In one, as a control, we introduced a premature stop codon (41 amino acids into the scFv) to form a truncated protein (Figure 1a). In the other, we used the wild-type sequence except for a previously described mutation that changes the aspartic acid at residue 31 in CDRI of the 3E10 heavy chain to asparagine (D31N) (Figure 1a). This has been shown to increase 3E10 scFv’s DNA binding and cellular penetration [30]. The scFv constructs were expressed and affinity purified from human 293 cells.

Numerous studies have investigated the incidence of PTEN mutations in patient derived glioma tissues and cell lines and have shown that PTEN expression is often lost in these samples [9]. As a model system to explore synthetic lethality with 3E10 in PTEN deficient cells, we used the U251 glioma cell line and a PTEN complemented isogenic cell line (U251+PTEN.) In addition, immortalized human astrocytes served as a control cell line. The PTEN protein expression status was determined *via* Western blot (Figure 1b).

To confirm cellular uptake of the scFv proteins, we treated the U251, U251+PTEN and astrocyte cell lines with each of the purified scFv’s by simple addition to the culture medium, followed by immunofluorescence microscopy using an antibody specific to the MBP tag. The 3E10 scFv purified protein was able to penetrate cells and localize primarily to the cytoplasm (Figure 1c). However, the truncated purified protein was unable to penetrate cells (Figure 1c), both consistent with our previously published work [23].

Clonogenic survival assays were then performed with the D31N or the truncated scFv in all three cell lines. Cells were pretreated with the scFv proteins for 24 hours before reseeding at low density for colony formation. Treatment with the D31N 3E10 scFv significantly reduced cell survival of the PTEN-deficient U251 cell line by approximately 50% at 1 and 2 µM concentrations (Figure 1d). It had no effect on the survival of either the U251+PTEN cells or the PTEN wild-type astrocytes, both of which express the PTEN protein. To note, increasing the concentration of the 3E10 scFv protein did not confer further effect on the U251 cells (Supplementary Figure 1a). As a control, treatment with the truncated scFv had no effect on survival of any of the cells (Figure 1d).

To directly measure cytotoxicity after treatment with the purified scFv proteins, cells were stained with Hoechst and propidium iodide at 3 and 7 days in order to quantify cell death and cell proliferation over time. Cells were treated for three days with the purified scFv proteins and then allowed to recover in fresh media (Figure 1e). As with the clonogenic survival assays, treatment with the D31N 3E10 did not affect cell death of either the U251+PTEN cells or astrocytes compared to the truncated construct or the buffer control, as both these lines express PTEN protein. However, treatment with the D31N scFv resulted in increased cell death in the PTEN-deficient U251 cell line at both time points, even days after replacement of media on day 3 with media not containing antibody (Figure 1f). We hypothesized that cell death was induced *via* apoptosis, and performed immunofluorescence staining to evaluate the presence of cleaved caspase-3 (Supplementary Figure 1b). Treatment with the D31N 3E10 did not induce apoptosis in the U251+PTEN cells, consistent with the PI staining assay results in Figure 1e. However, three-day treatment with the D31N 3E10 led to a 2.4-fold increase in apoptosis in U251 cells as compared to cells treated with the truncated control, with 46% of cells treated with 3E10 showing cleaved caspase-3 positive staining.

The cell proliferation assay also showed a large differential effect on the U251 cells *versus* the PTEN proficient U251+PTEN and astrocyte cells. (Figure 1g and Supplementary Figure 1c and 1d). Over time, there was also a detectable reduction in proliferation caused by 3E10 even in the PTEN proficient cells. This effect would be consistent with a general impact on RAD51 function in replication fork progression. Notably, U251 cells treated with the 3E10 scFv protein did not recover after the scFv was removed on day 3.

Our previous work demonstrated that 3E10 scFv physically interacts with RAD51 both *in vitro* based on studies with purified proteins and in cells based on co-IPs. We found that this physical interaction in cells leads to a sequestering of RAD51 in the cytoplasm and an inhibition of RAD51 foci formation in the nucleus upon ionizing radiation [23]. We therefore hypothesized that the 3E10 scFv protein’s ability to inhibit RAD51 localization at sites of DNA damage would increase cellular stress, ultimately resulting in the accumulation of DNA damage. Consistent with this, 1 day treatment with 3E10 scFv led to an increase in markers of DNA damage in U251 cells, both *via* ɣH2Ax foci (Supplementary Figure 2a and 2b) and p53BP1 foci (Supplementary Figure 2c and 2d). The 3E10 scFv did not induce increases in ɣH2Ax or p53BP1 foci formation in either of the PTEN-proficient cell lines (the U251+PTEN or astrocytes). Treatment with the truncated scFv, did not induce DNA repair foci formation in any of the cell lines.

Micronucleus formation is seen in cells with high basal levels of genomic instability due to genetic defects in DSB repair pathways [31-34], in irradiated cells [35], or in cells treated with chemical agents to induce replication stress [36]. Previous reports suggest that the PTEN-RAD51 signaling axis acts to mitigate replication stress and genomic instability [24]. PTEN deficient cells are known to have persistent baseline DNA damage and higher levels of micronuclei as compared to PTEN wild type cells [24, 37]. We therefore hypothesized that treatment with 3E10 in PTEN deficient cells would lead to a further increase in micronuclei as this signaling axis would be disrupted on both ends through the genetic loss of PTEN and the inhibition of RAD51 by 3E10.

**Figure 2:**
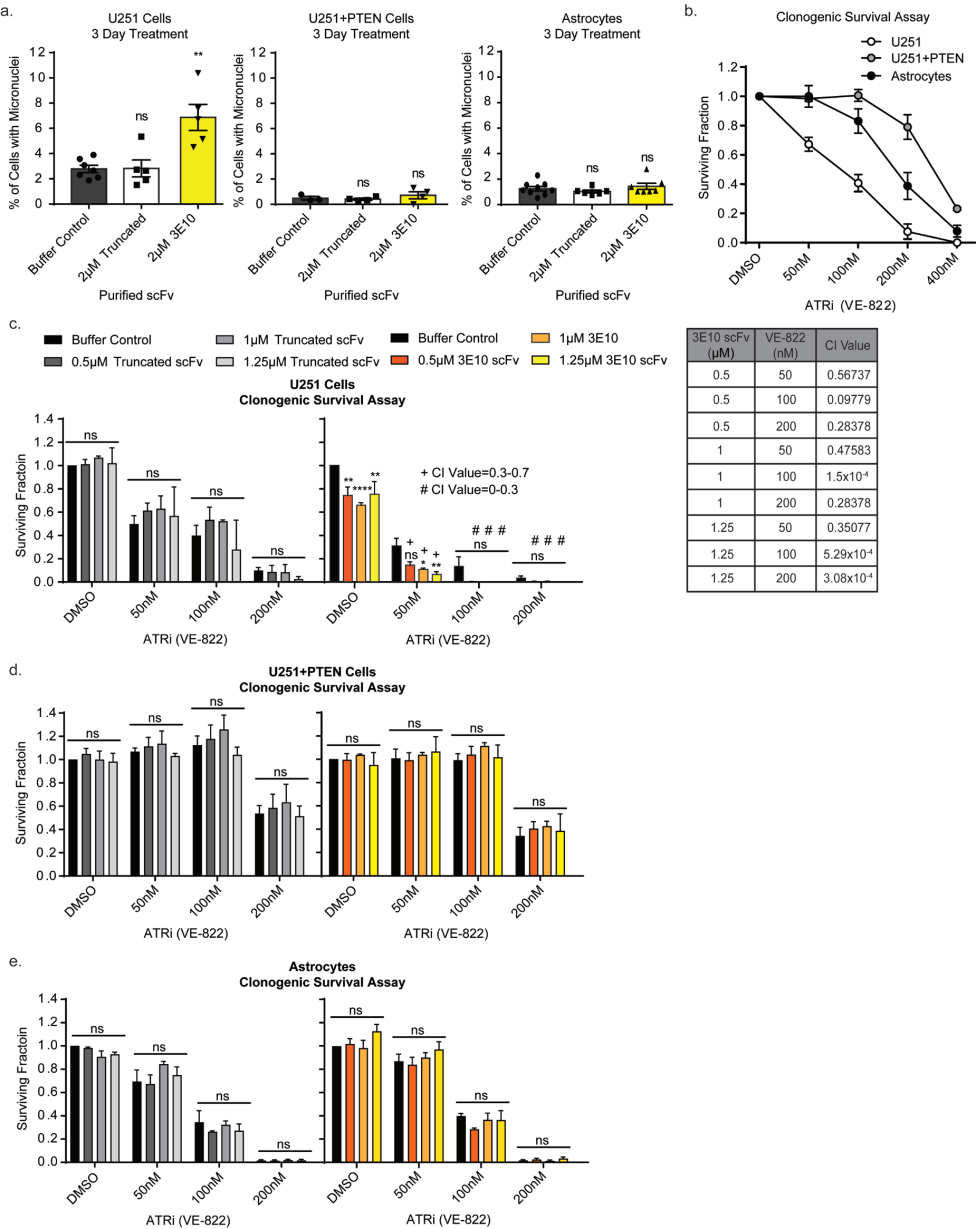
3E10 scFv induces persistent DNA damage which confers synergism with an ATR inhibitor **a.** U251, U251+PTEN or astrocyte cells were treated with purified 2XMBP-scFv proteins for three days. The percent of cells with micronuclei was plotted for each treatment condition for each cell line. Each data point symbol represents the mean for each experimental replicate. Error bars represent the SEM; **P < 0.01 by unpaired *t*-test. **b.** Clonogenic survival assay results to determine the optimal dose range of the ATR inhibitor. Cells were seeded at low density into the ATR inhibitor. After 24 hours, the drug was removed and cells were allowed to grow in fresh media in order to assess colony formation. Error bars represent the SEM. **c.**-**e.** Clonogenic survival assays to interrogate synergism between 3E10 scFv and an ATR inhibitor. U251 cells (c), U251+PTEN cells (d) or astrocytes (e) were pretreated with the purified scFvs for 24 hours before reseeding at low density into the ATR inhibitor. After 24 hours, the drug was removed and cells were allowed to grow in fresh media in order to assess colony formation. Error bars represent the SEM; ****P < 0.0001, **P < 0.01, and *P < 0.05 by unpaired *t*-test. Synergism was assessed using the CompuSyn software.

We interrogated the formation of micronuclei, indicative of persistent DNA damage through replication, in cells treated with 3E10 [38, 39]. To note, the PTEN-deficient U251 cells had a higher baseline level of micronuclei as predicted by the literature [24, 37]. Three-day treatment with 3E10 scFv induced additional micronuclei formation in the U251 cells, while having no effect on the U251+PTEN or astrocyte cell lines (Figure 2a). Treatment with the truncated scFv did not induce micronuclei formation in any of the cell lines. This data supports the idea that PTEN deficient cells that survive after treatment with 3E10 have enhanced replication stress resulting in micronuclei and are poised for mitotic catastrophe.

### 3E10 is synergistic with an ATR inhibitor in PTEN deficient glioma cells

ATR signaling has been shown to play a key role in replication as ATR is activated upon replication fork stalling [40] and acts to protect stalled replication forks and suppress global origin firing [41]. ATR has been a promising target for cancer therapy as proliferating cancer cells often have higher levels of replication stress and absent G1 checkpoints, making cancer cells reliant on ATR signaling [40]. Inhibition of ATR in cancer cells has been shown to generate excessive ssDNA at stalled replication forks which exhausts cellular RPA and leads to mitotic catastrophe [41].

We chose to inhibit ATR *via* a small molecule inhibitor (VE-822) and assess synergism with 3E10 scFv in PTEN deficient cells in an effort to induce mitotic catastrophe and thus enhance the cytotoxicity of 3E10 in PTEN deficient cells. Clonogenic survival assays were first performed to interrogate the sensitivity of all three cell lines to the small molecule inhibitor of ATR. We found that PTEN expression of each cell line (Figure 2b) was inversely correlated with sensitivity to the ATR inhibitor: U251 cells lacking PTEN expression had the highest sensitivity, while the PTEN complemented U251 cells were the least sensitive, and the immortalized astrocytes had an intermediate sensitivity to the ATR inhibitor. Parallel effects were seen in cell proliferation and cell death assays (Supplementary Figure 3a and 3b).

Clonogenic survival assays were then performed in cells treated with or without the ATR inhibitor in combination with D31N 3E10 scFv, the truncated scFv, or buffer control, at the indicated doses (Figure 2c and 2d). Cells were pretreated with the scFv proteins or buffer alone for 24 hours before reseeding at low density in the presence of the ATR inhibitor. Cells were left in the ATR inhibitor at low density for 1 day before the media was replaced. Pre-treatment with the truncated scFv had no effect on any of the cell lines’ sensitivity to the ATR inhibitor. However, pre-treatment of the U251 cells with the 3E10 scFv synergized with the ATR inhibitor at all three doses. Pre-treatment of either the U251+PTEN or astrocyte cells with the 3E10 scFv had no effect on these cell lines’ sensitivities to the ATR inhibitor (Figures 2c and 2d).

### D31N 3E10 scFv is synthetic lethal with PTEN deficiency in patient derived melanoma cells

**Figure 3:**
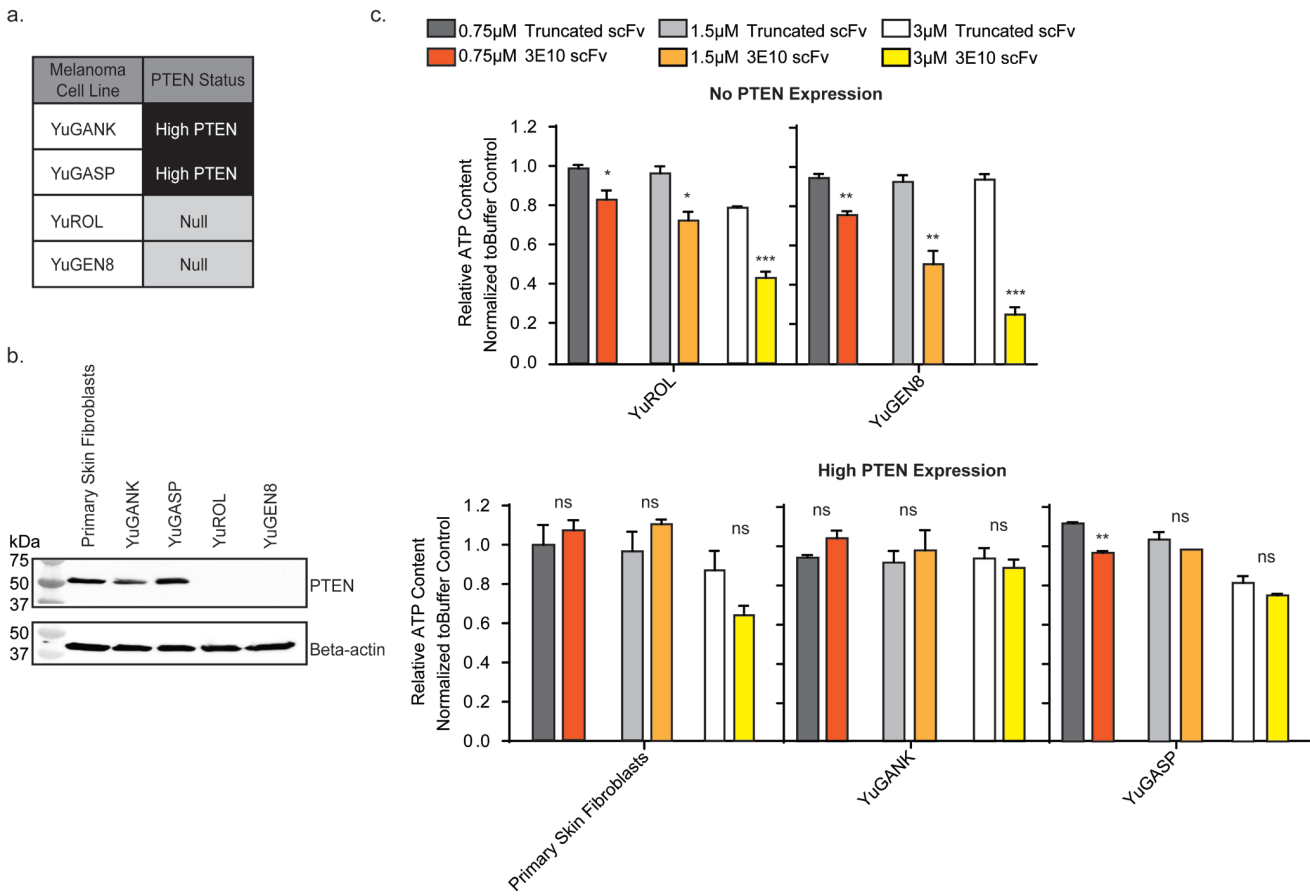
3E10 scFv decreases cell viability in PTEN deficient melanoma cells **a.** Summary of PTEN status of four melanoma cell populations from the Specimen Resource Core of the Yale SPORE in Skin Cancer. **b.** Western blot analysis of PTEN expression in the melanoma cells and normal primary human skin fibroblast cells. **c.** CellTiter-Glo luminescent cell viability assay results. Error bars represent the SEM; ***P < 0.001, **P < 0.01, and *P < 0.05 by unpaired *t*-test.

PTEN is frequently mutated or PTEN expression is lost in melanomas [2, 4, 5, 42]. We obtained patient derived, primary melanoma cell populations from the Specimen Resource Core of the Yale SPORE in Skin Cancer [18, 43-46]. Two high-PTEN expressing melanoma populations (YuGANK and YuGASP) were chosen in comparison to two PTEN-null populations (YuROL and YuGEN8) for analysis (Figure 3a). Primary human skin fibroblasts were also used as a control. The PTEN protein expression status of these five cell populations was determined *via* Western blot (Figure 3b). The melanoma cell populations chosen for these studies do not adequately form colonies. Therefore, the CellTiter-Glo luminescent cell viability assay was performed in lieu of the clonogenic survival assay. Cells were treated for five days with the D31N scFv or the truncated scFv at increasing concentrations, and viability was assessed (Figure 3c). The D31N 3E10 scFv did not consistently have a statistically significant impact on the viability of the PTEN expressing cells (normal skin fibroblasts, YuGANK, or YuGASP) as compared to treatment with the truncated scFv. However, the purified 3E10 scFv protein significantly decreased cell viability in the PTEN deficient YuROL and YuGEN8 cells in a dose dependent manner.

**Figure 4:**
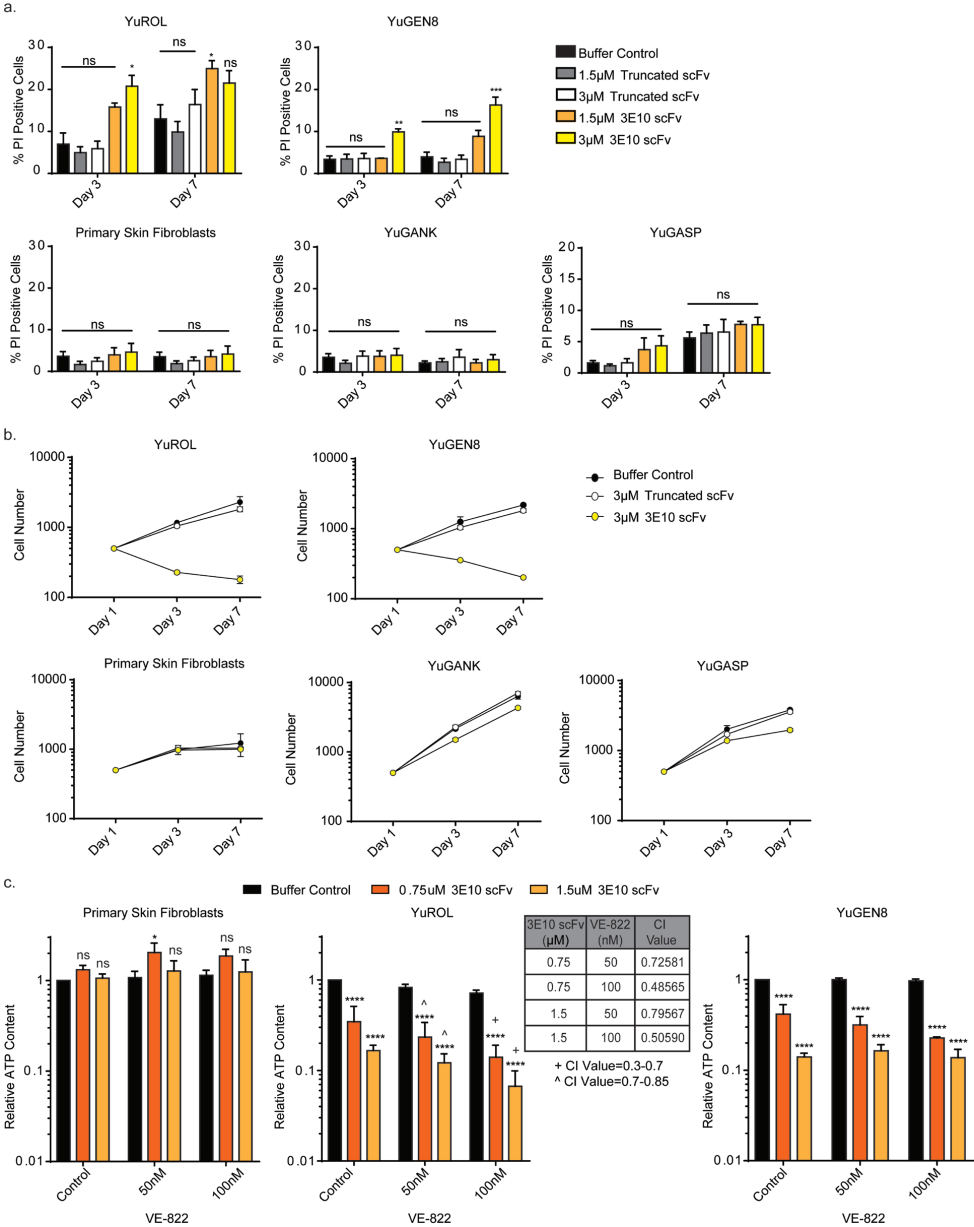
3E10 induces cell death and delays proliferation in PTEN deficient melanoma cells, and these effects confer synergism with an ATR inhibitor **a.** Cell death for each cell population under each treatment condition was plotted for each time point. Error bars represent the SEM; ***P < 0.001, **P < 0.01 and *P < 0.05 by unpaired *t*-test. **b.** Cell number for each cell type under each treatment condition was plotted for each time point. Error bars represent the SEM. **c.** CellTiter-Glo luminescent cell viability assay results to interrogate synergism between 3E10 scFv and an ATR inhibitor. Cells were pretreated with the purified 3E10 scFv for 24 hours before the addition of the ATR inhibitor. Error bars represent the SEM; ****P < 0.0001 and *P < 0.05 by unpaired *t*-test. Synergism was assessed using the CompuSyn software.

To directly measure cytotoxicity after treatment with the purified scFv proteins, cells were stained with Hoechst and propidium iodide at different time points in order to quantify cell death and cell proliferation over time, as done with the glioma and astrocyte cell lines. Cells were treated for three days with the scFv proteins and then allowed to recover in fresh media. Treatment with the scFvs did not affect cell death in the PTEN-proficient cells (normal primary skin fibroblasts, YuGANK, or YuGASP). However, three-day treatment with the D31N scFv protein increased cell death in the PTEN-deficient YuROL and YuGEN8 cells. Further, even after removal of D31N scFv, cell death continued to occur over time in the YuROL and YuGEN8 cells. Treatment with the truncated purified scFv protein, which is unable to penetrate cells, did not affect cell survival in any of the cell populations (Figure 4a). The D31N 3E10 scFv also substantially reduced to cell proliferation in the PTEN-deficient YuROL and YuGEN8 cells, while having a slight effect on the YuGANK and YuGASP cells (Figure 4b and Supplementary Figure 4). Importantly, there was no effect on cell proliferation in the primary skin fibroblasts at either dose.

### 3E10 is synergistic with an ATR inhibitor in PTEN deficient patient derived melanoma cells

Potential synergism between the ATR inhibitor (VE-822) and the non-truncated 3E10 scFv in the patient derived cells was also assessed. Cell proliferation and cell death assays were first performed to interrogate the sensitivity of the cells to the small molecule inhibitor of ATR. The cell proliferation of the PTEN-deficient YuROL and YuGEN8 cells was drastically affected, while minimal effect was observed in the PTEN-proficient cells (Supplementary Figure 5). Further, no increase in cell death was observed in any cell population, suggesting that the ATR inhibitor (VE-822) mainly impacts cell proliferation in these cells (Supplementary Figure 5).

Cell viability assays were then performed with the scFvs. Cells were pretreated with the scFv proteins for 24 hours before the addition of the ATR inhibitor. Pre-treatment with the truncated scFv had no effect on any of the cells’ sensitivity to the ATR inhibitor (Supplementary Figure 6). Pre-treatment of the primary skin fibroblasts with the 3E10 scFv had also no effect on these cells sensitivities to the ATR inhibitor (Figure 4c). However, pre-treatment of the YuROL cells with the 3E10 scFv synergized with the ATR inhibitor at both doses (Figure 4c). Although the 3E10 scFv sensitized the YuGEN8 cells to the ATR inhibitor (most notably at the 0.75µM dose of the 3E10 scFv), no synergism was observed. Because melanoma is characterized by a high mutational burden and a genetic landscape [47], further investigation is warranted to determine which genetic sub-type types of melanoma may benefit from a combination approach such as is described here.

## DISCUSSION

PTEN is the second most frequently mutated or inactivated tumor suppressor gene in human cancers, with PTEN alterations found in approximately 8% of all human cancers [48, 49] and in 15% of melanomas (TCGA), making novel therapeutic approaches to treat these cancers a priority. When PTEN is lost or its nuclear import impaired, genomic instability has been observed, providing an exploitable therapeutic window for PTEN-null cancers, which has been the basis for clinical trials investigating the effect of PARP inhibitors in participants with mutations or deletions in PTEN leading to functional PTEN loss.

Here, through cell-based assays, we found that 3E10 affects cellular viability of PTEN deficient cells, indicating that inhibiting HDR with 3E10 leads to cytotoxicity in PTEN deficient cells. This cytotoxicity is likely due to the fact that PTEN deficient cells are both deficient in NEHJ [18] and have a significant baseline burden of DNA damage and replication stress that is exacerbated with treatment with 3E10, as indicated by the induction of ɣH2Ax and p53BP1 foci and the occurrence of micronuclei after treatment with 3E10 scFv.

Further, the cellular and replicative stress induced by 3E10 in PTEN-deficient cells sensitizes these cells to a small molecule inhibitor of ATR. We have extended these findings to a clinically relevant model of patient derived primary melanoma cell populations. As such, these results may provide the basis for further pre-clinical development to devise targeted synthetic lethal strategies in PTEN deficient cancers, and to further aid in guiding the design of new personalized cancer therapies.

## MATERIALS AND METHODS

### Cloning of 3E10 scFv and site directed mutagenesis

The single chain variable fragment of 3E10 was cloned into a phCMV1_2XMBP expression vector. Point mutations were introduced into regions of the scFv using the QuikChange Site-Directed Mutagenesis Kit (Stratagene). The plasmid was sequenced to ensure no undesired mutations were introduced.

### Purification of 2XMBP-scFvs

Purification of the 2XMBP-scFv proteins was performed as previously described [23]. Briefly, 2XMBP-scFv expression constructs were transfected into suspension Expi293F Cells (Life Technologies, grown at 37°C and 5%CO2) with polyethylenimine (PEI linear, MW ∼25,000 from Polysciences, Inc.). BalanCD CHO FEED 3 (Irvine Scientific) and Valproic acid sodium salt (Sigma-Aldrich, 3.8mM final concentration) were added each of the next two days to enhance expression. The cells were grown at for three days after transfection and then were harvested by centrifugation. The cell pellet was resuspended in lysis buffer (50mM HEPES pH 7.5, 250mM NaCl, 1% NP-40, 1mM MgCl2, 1x Protease Inhibitor Cocktail (Roche complete, EDTA-free), 1mM DTT). The lysate was cleared by centrifugation at 10,000 rpm for 15 minutes and then incubated with amylose resin (New England BioLabs, Inc.) overnight at 4°C. The lysate was spun down and the supernatant was removed. The amylose beads were washed three times with wash buffer (50mM HEPES pH 7.5, 250mM NaCl, 0.5mM EDTA, 1mM DTT). The bound protein was eluted from the amylose beads in wash buffer containing 10mM maltose. The eluate containing the 2XMBP-scFv was buffer exchanged into DMEM media.

### Western blots for PTEN

Cells in culture were collected *via* trypsinization and pelleted *via* centrifugation. Cell pellets were lysed in AZ lysis buffer (50mM Tris pH 8, 250mM NaCl, 1% NP-40, 0.1% SDS, 5mM EDTA, 10mM Na4P2O7, 10mM NaF, 1x cOmplete EDTA-free Protease Inhibitor Cocktail (Roche), 1x PhosSTOP (Roche)). The protein concentration of each sample was determined using the DC™ (detergent compatible) protein assay (Bio-Rad Laboratories, Inc.). Protein concentrations were normalized and samples were prepared with 5x Laemmli sample buffer. Samples were run on a gradient gel (Bio-Rad) and transferred for Western blot on 0.45um Nitrocellulose membrane (Bio-Rad).

The primary antibodies used were mouse anti-PTEN (sc7974, Santa Cruz Biotechnology), and mouse anti-Beta Actin (Cell Signaling Technology #4970). Primary antibodies were used at 1:1000 dilutions and were incubated for 1 to 2 hours at room temperature or overnight at 4°C. Secondary goat anti-mouse antibody (Thermo Fisher Scientific/Pierce) or were used at a 1:10,000 dilution for 1 hour at room temperature. Primary and secondary antibodies were prepared in 5% milk. Three washes with Tris Buffered Saline with Tween 20 were each performed after primary incubation and after secondary incubation. Membranes were developed using SuperSignal West Pico Chemiluminescent Substrate (Thermo Fisher Scientific).

### Immunofluorescence - cell penetration of purified 2XMBP-scFv

U251, U251+PTEN or Astrocyte cells were treated with purified 2XMBP-scFv proteins for 24 hours in chamber well slides (Millipore Millicell EZ Slides). Cells were fixed with 1% paraformaldehyde/2% sucrose for 15 minutes at room temperature, followed by 100% methanol for 30 minutes at -20°C, and 50% methanol/50% acetone for 20 minutes at -20°C. Slides were then incubated in permeabilization/blocking solution (10% BGS, 0.5% Triton X-100 in phosphate-buffered saline (PBS)) at room temperature for 1 h. Primary antibody (mouse anti-MBP monoclonal antibody, New England BioLabs, Inc. #E8032S) was diluted 1:500 in permeabilization/blocking solution and used to stain cells at 4°C overnight. The secondary antibody used was Alexa Fluor 594-conjugated goat anti-mouse immunoglobulin G (IgG) (Life Technologies). Three washes with PBS with Triton X-100 and four washes with PBS were each performed after primary incubation and after secondary incubation. Cells were costained with DAPI to visualize the nuclei. Slides were imaged on a Zeiss-CARV II confocal microscope.

### Clonogenic survival assays

U251, U251+PTEN or Astrocyte cells seeded into 6 well plates at 30,000 cells/well and were pretreated with purified 2XMBP-scFv proteins for 24 hours. The following day, the cells were reseeded in triplicate into 6 well plates at low density (500 cells/well for U251 and U251+PTEN cells and 1,000 cells/well for Astrocytes) into fresh media or a small molecule inhibitor of ATR (VE-822, Selleckchem #S7102) for 24 hours before the media containing the ATR inhibitor was replaced with fresh media. Cells were cultured for 1 to 2 weeks until colonies had formed. Cells on the 6 well plates were permeabilized with 0.9% saline solution and then stained with crystal violet in 80% methanol. Colonies of ≥ 50 cells were then quantified.

Synergism was calculated using the CompuSyn software (ComboSyn, Inc.).

### Propidium iodide staining assays for cell proliferation and cell death quantifications

Cells were seeded into 96 well plates at 500 cells/well and were treated with purified 2XMBP-scFv proteins for 72 hours. The media containing purified 2XMBP-scFv proteins was removed and replaced with media containing PI/RNase staining buffer (BD Biosciences) and Hoechst dye (Thermo Fisher). The plates were imaged and the nuclei were quantified using a Cytation3 Cell Imaging Multi-Mode Reader (BioTek). The media was then replaced with fresh media and cells were cultured for another 96 hours. Media was then replaced with media containing PI/RNase staining buffer (BD Biosciences) and Hoechst dye (Thermo Fisher). The plates were imaged again and the nuclei were quantified using a Cytation3 Cell Imaging Multi-Mode Reader (BioTek). Total cell number was quantified based on Hoechst staining. Percent cell death was quantified from the PI positive cell number divided by total cell number. Experiments were performed at least three times for biological replicates. Statistical significance was determined using the unpaired t test (GraphPad Prism).

### Micronuclei quantifications

U251, U251+PTEN or Astrocyte cells were treated with purified 2XMBP-scFv proteins for three days. Nuclei were stained with Hoechst dye (Thermo Fisher) and the cells were imaged using an EVOSfl Digital Inverted Microscope (Fisher Scientific). Images were visualized and quantified using ImageJ.

Experiments were performed at least three times for biological replicates. Statistical significance was determined using the unpaired t test (GraphPad Prism).

### Immunofluorescence - DNA repair foci quantifications or cleaved caspase 3

U251, U251+PTEN or Astrocyte cells were treated with purified 2XMBP-scFv proteins for 24 hours in chamber well slides (Millipore Millicell EZ Slides). Cells were fixed as above and incubated in permeabilization/blocking solution (as above) at room temperature for 1 h. Primary antibodies were diluted 1:500 in permeabilization/blocking solution and used to stain cells at 4°C overnight. The primary antibodies used were rabbit anti-p-Histone H2A.X Serine 139 (Cell Signalling Technologies #9718), rabbit anti-p-53BP1 Serine 1778 (Cell Signalling Technologies #2675), and rabbit anti-cleaved caspase-3 (Cell Signalling Technologies #9661). The secondary antibody used was Alexa Fluor 488-conjugated goat anti-rabbit immunoglobulin G (IgG) (Life Technologies). Three washes with PBS with Triton X-100 and four washes with PBS were each performed after primary incubation and after secondary incubation. Cells were co-stained with DAPI to visualize the nuclei. Slides were imaged on a Zeiss-CARV II confocal microscope. Images were visualized and quantified using ImageJ. Statistical significance was determined using the unpaired t test (GraphPad Prism).

### Cell viability assay

Cells were seeded at 500 cells per well in a 96 well plate and then treated with purified 2XMBP-scFv proteins, a small molecule inhibitor of ATR (VE-822, Selleckchem #S7102), or both the purified 2XMBP-scFvs and ATR inhibitor. For the combination of the purified 2XMBP-scFvs and ATR inhibitor, cells were pre-treated with the purified 2XMBP-scFvs for 24 hours and then the ATR inhibitor was added. Cells were treated for five days before cell viability was assessed *via* the CellTiter-Glo Luminescent Cell Viability Assay (Promega) according to the manufacturer’s instructions. Experiments were performed at least two times for biological replicates. Statistical significance was determined using the unpaired t test (GraphPad Prism). Synergism was calculated using the CompuSyn software (ComboSyn, Inc.).

## SUPPLEMENTARY MATERIALS FIGURES


