# Supplementary material for: Synthetic lethality of a cell-penetrating anti-RAD51 antibody in PTEN-deficient melanoma and glioma cells
Source: Oncotarget. 2019 Feb 12;10(13):1272–83. doi: 10.18632/oncotarget.26654 (PMC6407680; doi:10.18632/oncotarget.26654)
Supplement: Supplementary file 1 [file oncotarget-10-1272-s001.pdf]

Synthetic lethality of a cell-penetrating anti-RAD51 antibody in PTEN-deficient melanoma and glioma cells

SUPPLEMENTARY MATERIALS

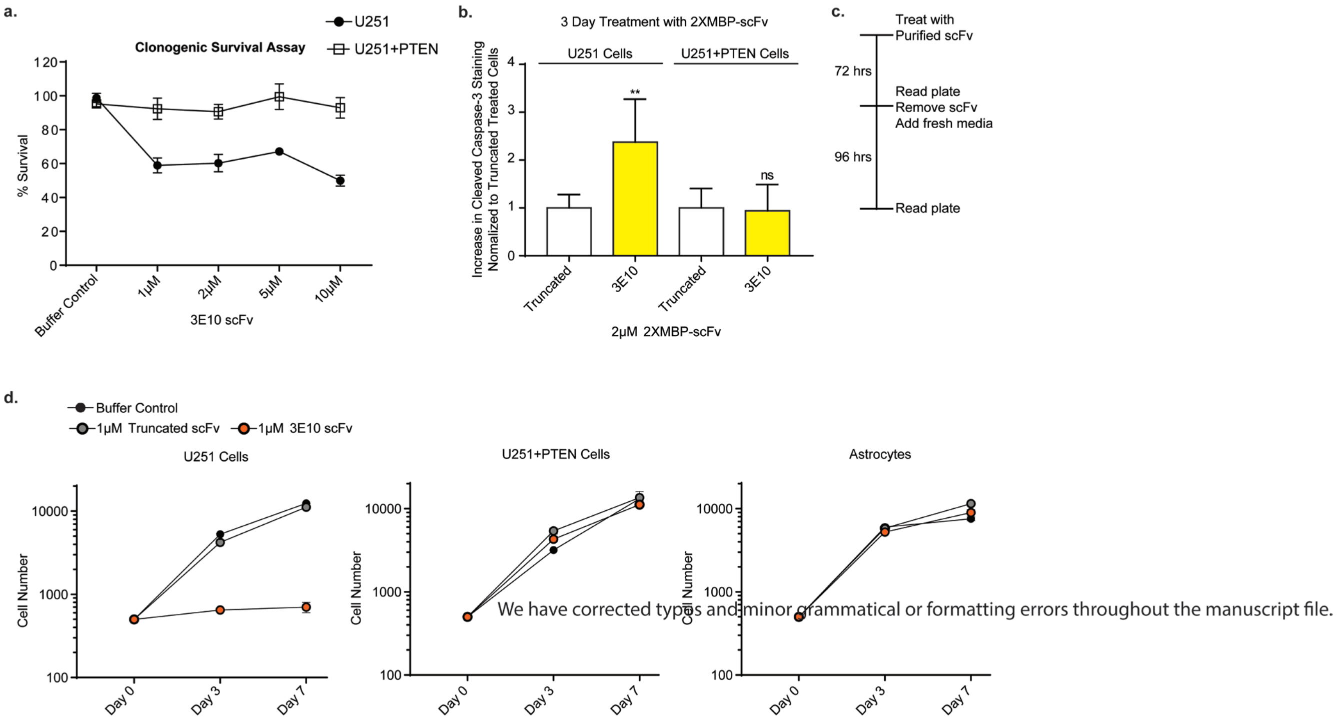

We have corrected typos and minor grammatical or formatting errors throughout the manuscript file.

Supplementary Figure 1: 3E10 scFv confers synthetic lethality and delays proliferation in PTEN-deficient U251 glioma cells. a. Clonogenic survival results of U251 and U251+PTEN cell lines treated with a dose range of purified 3E10-scFv protein. **b.** Immunofluorescence results for cleaved caspase-3 staining in U251 and U251+PTEN cells after three-day treatment with either truncated scFv or 3E10 scFv. Error bars represent the SD; $**P < 0.01$ by unpaired *t*-test. **c.** U251, U251+PTEN or astrocyte cell lines were treated with either truncated scFv or 3E10 scFv for three days and then grown in fresh media for an additional four days. On day 3 and day 7, cells were stained with Hoescht dye and propidium iodide in order to quantify cell number over time. **d.** Cell number for each cell line treated with 1uM scFv was plotted for each time point. Error bars represent the SEM.

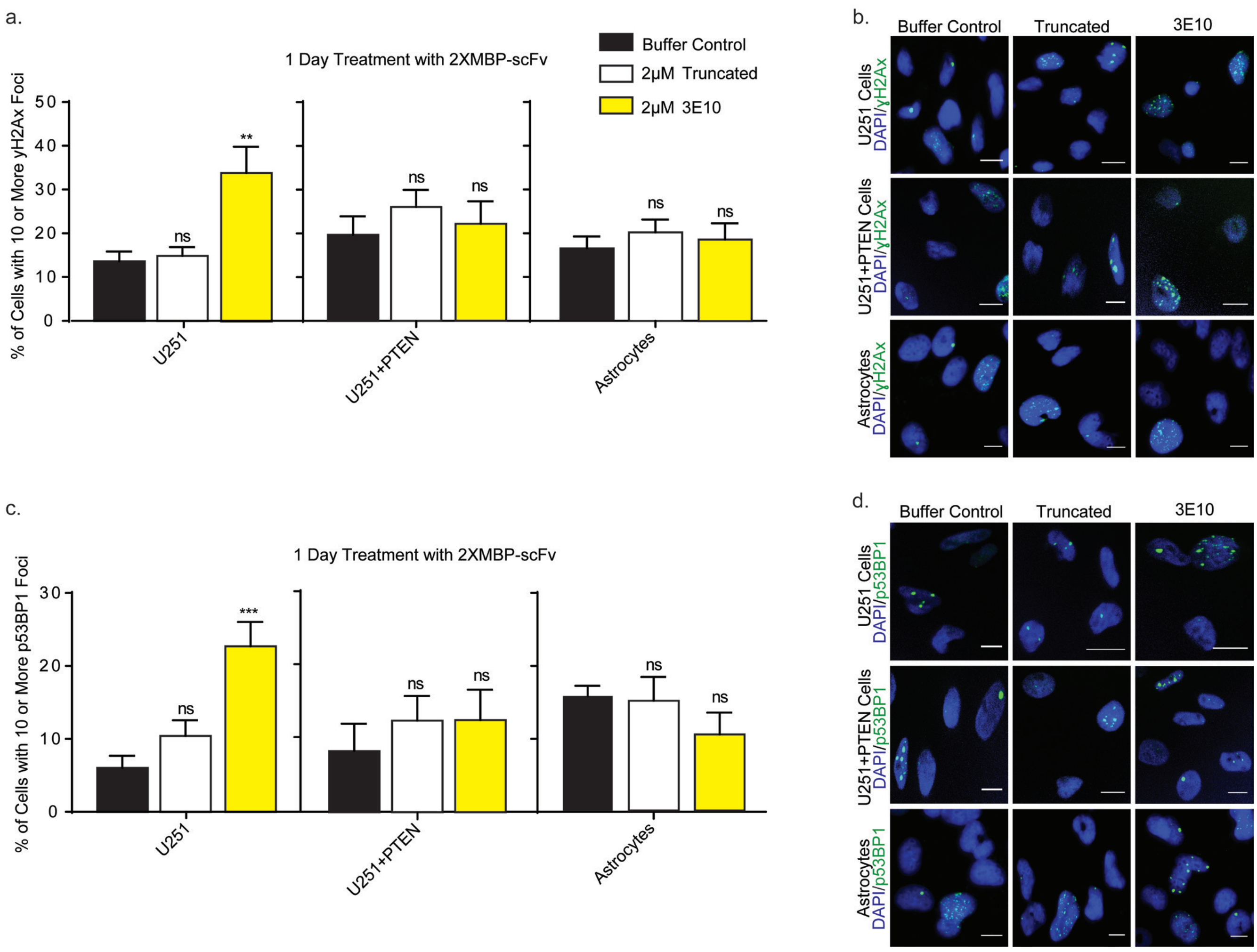

Supplementary Figure 2: Treatment with 3E10 scFv leads to increased levels of DNA damage. U251, U251+PTEN, or Astrocyte cells were treated with 2μM purified 2XMBP-scFv proteins for 24 hours. Cells were fixed and stained for immunofluorescence. The percent of cells with 10 or more ɣH2Ax **a.** or phospho-53BP1 **c.** foci was plotted for each treatment condition for each cell line. **b.** and **d.** Representative images. Error bars represent the SEM; ***$P < 0.001$ and **$P < 0.01$ by unpaired *t*-test.

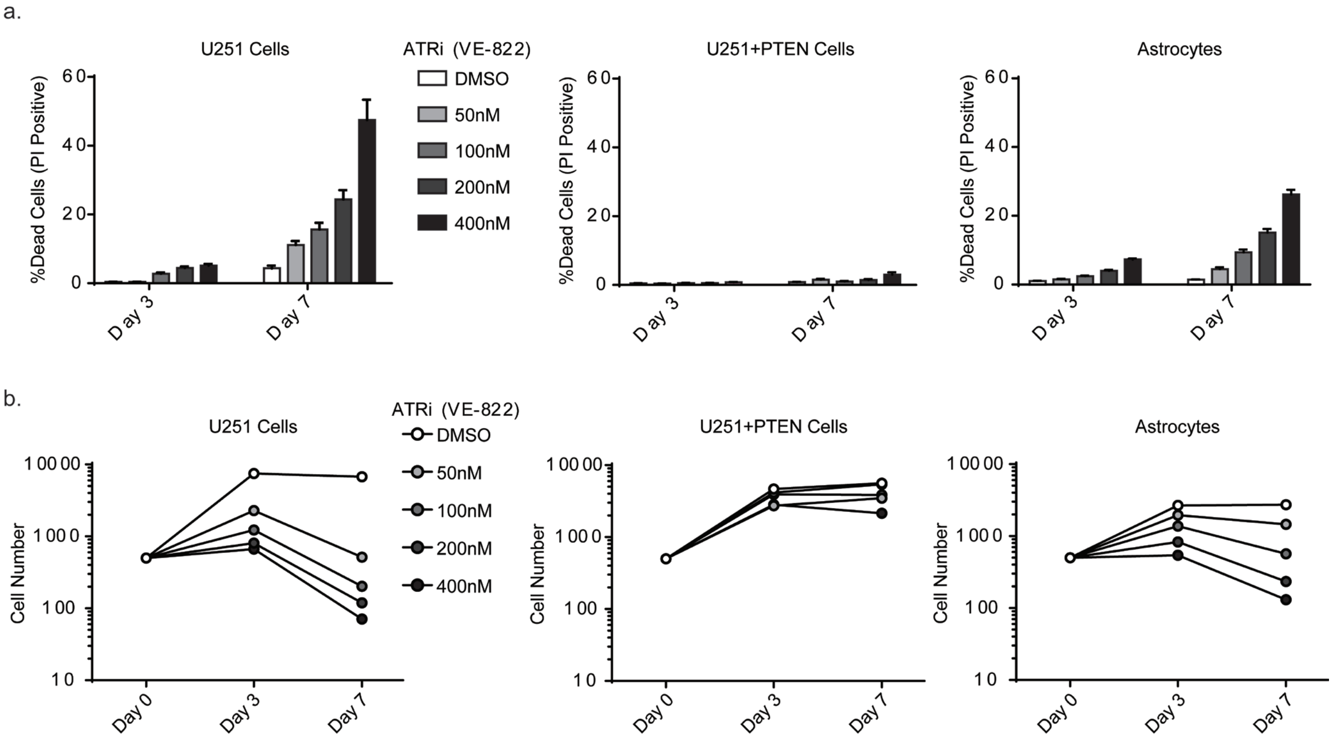

Supplementary Figure 3: Sensitivity to an ATR inhibitor correlates with PTEN expression. U251, U251+PTEN or astrocyte cell lines were treated with a dose range of an ATR inhibitor for three days and then grown in fresh media for an additional four days. On day 3 and day 7, cells were stained with Hoescht dye and propidium iodide in order to quantify cell death over time. **a.** Cell death for each cell line under each treatment condition was plotted for each time point. **b.** Representative cell number for each cell line under each treatment condition was plotted for each time point.

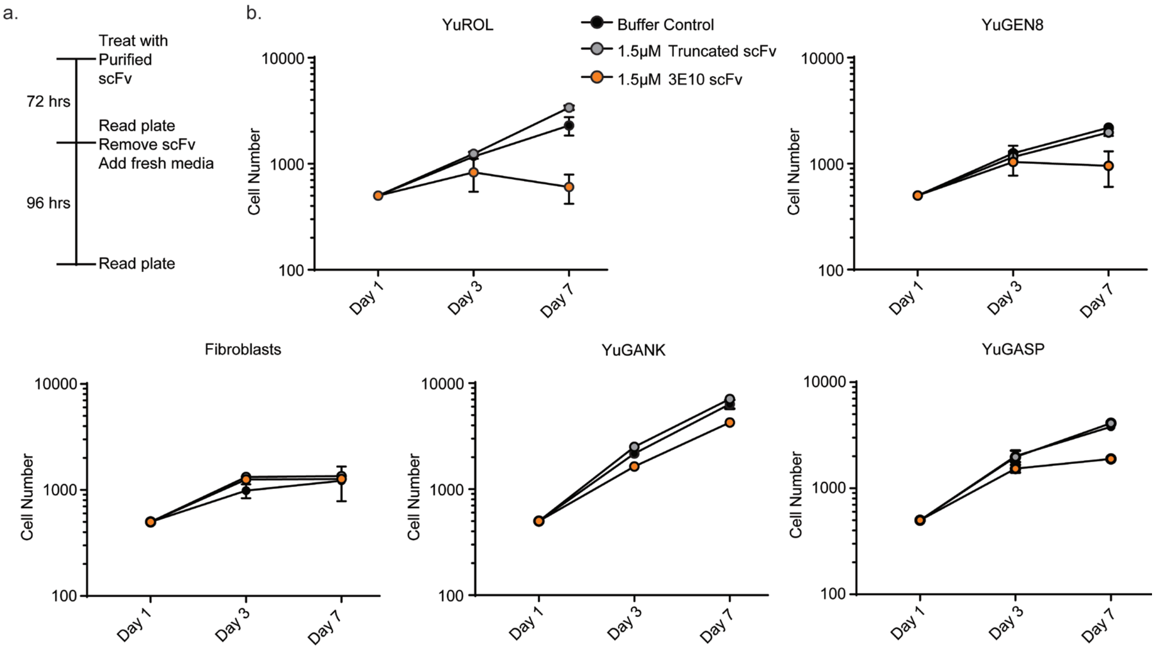

Supplementary Figure 4: 3E10 scFv delays proliferation in PTEN deficient melanoma cells. a. Melanoma cells or primary human skin fibroblasts were treated with either truncated scFv or 3E10 scFv for three days and then grown in fresh media for an additional four days. On day 3 and day 7, cells were stained with Hoescht dye and propidium iodide in order to quantify cell number over time. **b.** Cell number for each cell type treated with 1.5μM scFv was plotted for each time point. Error bars represent the SEM.

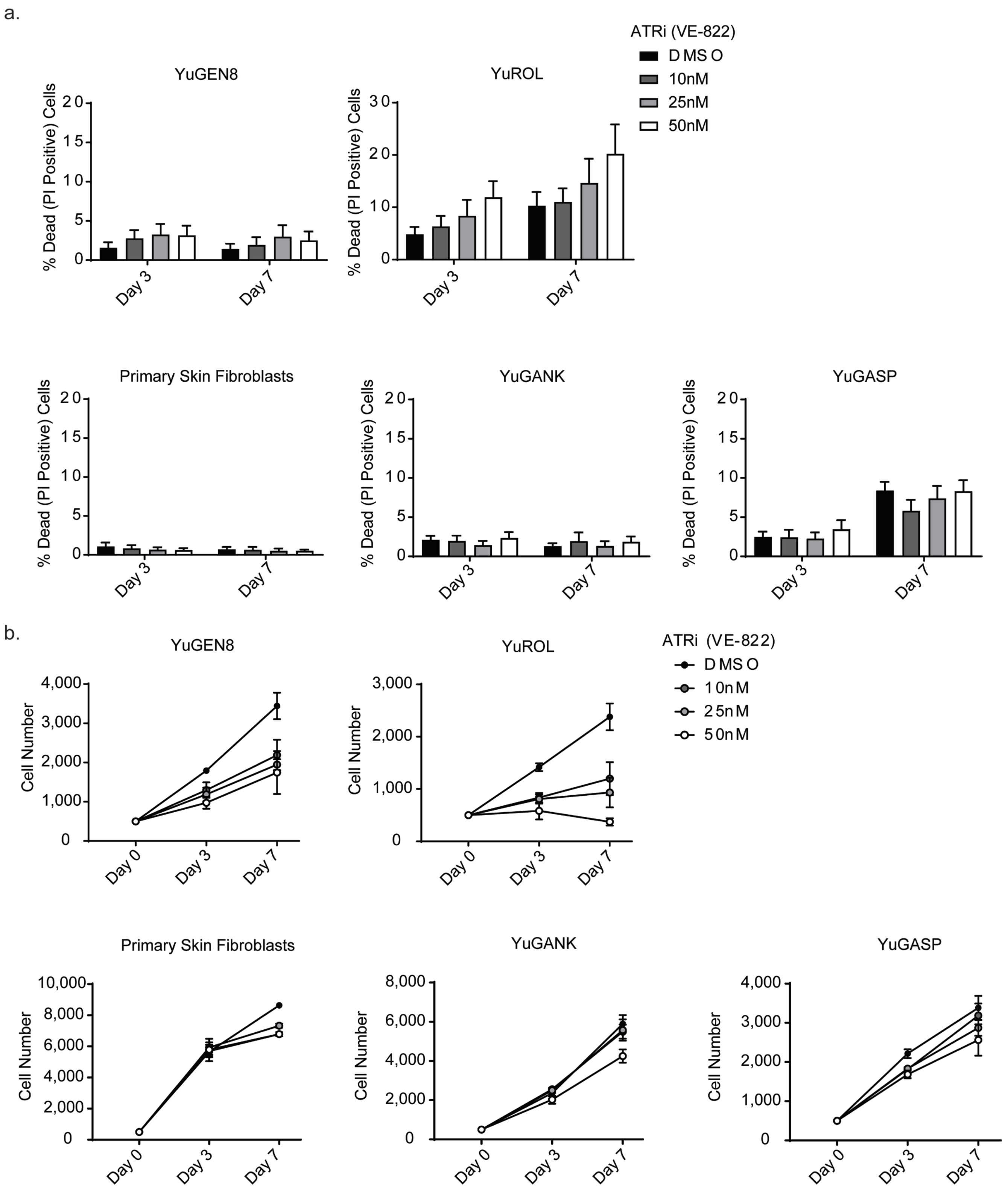

Supplementary Figure 5: An ATR inhibitor selectively delays proliferation in melanoma cells. Melanoma cells or primary human fibroblasts were treated with the ATR inhibitor for three days and then grown in fresh media for an additional four days. On day 3 and day 7, cells were stained with Hoescht dye and propidium iodide in order to quantify cell number and cell death over time. **a.** Cell death for each cell type under each treatment condition was plotted for each time point. Error bars represent the SEM. **b.** Cell number for each cell type under each treatment condition was plotted for each time point. Error bars represent the SEM.

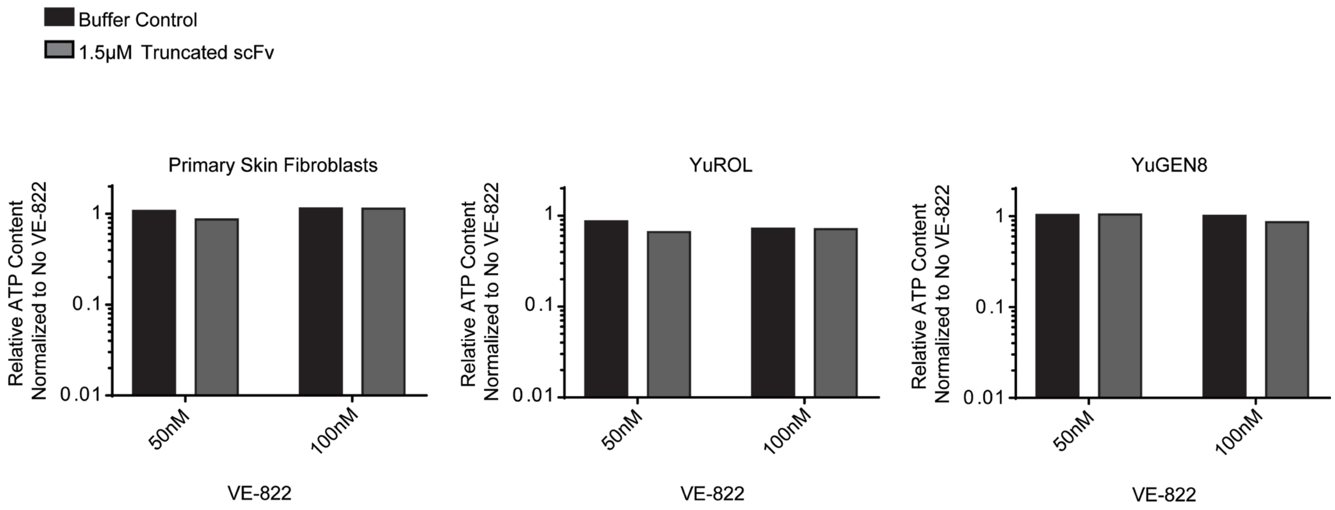

Supplementary Figure 6: Truncated 3E10 scFv does not synergize with an ATR inhibitor. A. CellTiter-Glo luminescent cell viability assay results to interrogate synergism between a high dose of truncated 3E10 scFv and an ATR inhibitor. Representative assay results are shown.
